# The impact of atrial fibrillation on oxygen uptake and haemodynamics in patients with heart failure: a systematic review and meta-analysis

**DOI:** 10.1093/ehjopen/oeaf003

**Published:** 2025-02-04

**Authors:** Veronika Schmid, Stephen J Foulkes, Jenelle K Dziano, Jing Wang, Jan Verwerft, Adrian D Elliott, Mark J Haykowsky

**Affiliations:** Technical University of Munich, School of Medicine and Health, Department for Preventive Sports Medicine and Sports Cardiology, TUM University Hospital, Georg-Brauchle-Ring 56, 80992 Munich, Germany; Integrated Cardiovascular Exercise Physiology and Rehabilitation Lab, Faculty of Nursing, College of Health Sciences, University of Alberta, 3-045 Edmonton Clinic Health Academy, 11405 87 Ave NW, Edmonton, Alberta, Canada T6G 1C9; Integrated Cardiovascular Exercise Physiology and Rehabilitation Lab, Faculty of Nursing, College of Health Sciences, University of Alberta, 3-045 Edmonton Clinic Health Academy, 11405 87 Ave NW, Edmonton, Alberta, Canada T6G 1C9; Heart, Exercise and Research Trials Lab, St Vincent’s Institute of Medical Research, 9 Princes Street, Fitzroy, Victoria 3065, Australia; Centre for Heart Rhythm Disorders, University of Adelaide & Royal Adelaide Hospital, The University of Adelaide, Port Rd, Adelaide, South Australia 5000, Australia; Division of Public Health, School of Medicine, University of Utah, 375 Chipeta Way, Salt Lake City, UT 84108, USA; Department of Cardiology, Jessa Hospital, Stadsomvaart 11, 3500 Hasselt, Belgium; Faculty of Medicine and Life Sciences, UHasselt, Agoralaan gebouw D, 3590 Diepenbeek, Belgium; Centre for Heart Rhythm Disorders, University of Adelaide & Royal Adelaide Hospital, The University of Adelaide, Port Rd, Adelaide, South Australia 5000, Australia; Integrated Cardiovascular Exercise Physiology and Rehabilitation Lab, Faculty of Nursing, College of Health Sciences, University of Alberta, 3-045 Edmonton Clinic Health Academy, 11405 87 Ave NW, Edmonton, Alberta, Canada T6G 1C9

**Keywords:** Atrial fibrillation, Heart failure, Exercise intolerance, Exercise haemodynamics, Peak oxygen uptake

## Abstract

**Aims:**

Atrial fibrillation (AF) may exacerbate exercise intolerance and haemodynamic limitations in individuals with heart failure (HF). Therefore, we performed a systematic search and meta-analysis to quantify the impact of AF on exercise tolerance (peak oxygen uptake, VO_2_peak; primary outcome) and exercise haemodynamics (secondary outcomes) in patients with HF.

**Methods and results:**

PubMed, Scopus, and Web of Science databases were systematically searched for articles from inception to June 2024. Studies were included if they: (i) examined participants with HF; (ii) compared participants with AF to those not in AF (i.e. sinus rhythm); (iii) measured VO_2_peak from expired gas analysis. A fixed effects meta-analysis was performed, with groups compared using the weighted average effect size, represented as the weighted mean difference (WMD) with 95% confidence intervals (95% CI). Of 573 identified studies, 16 met the full inclusion comparing VO_2_peak in HF-patients in AF [HF-AF; *n* = 1,271, 68% male, 67 years, left ventricular ejection fraction (LVEF): 41%], and HF in sinus rhythm (HF-SR; *n* = 4910; 62% male, 62 years, LVEF: 41%). VO_2_peak was significantly lower in HF-AF (WMD: −1.55mL/kg/min, 95%-CI: −1.81 to −1.28, *n* = 6471). This coincided with a slightly lower peak heart rate (WMD: −2.94 b/min, 95%-CI: −4.76 to −1.13 b/min, *n* = 5115), decreased O_2_pulse (WMD: −1.58 mL/beat, 95% CI: −1.90 to −1.26, *n* = 3049), and lower systolic blood pressure (WMD: −11.11 mmHg, 95% CI: −14.01 to −8.21, *n* = 2409).

**Conclusion:**

In patients with HF, AF is associated with greater VO_2_peak impairment, potentially due to reduced stroke volume and/or arterio-venous oxygen difference. This highlights the importance of combined strategies to identify and manage AF in individuals with HF.

## Graphical abstract

**graphical abstract:**
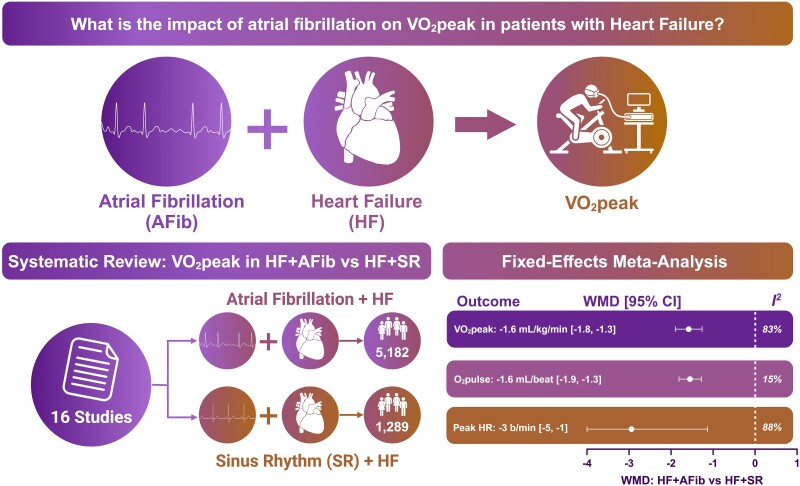

## Introduction

Reduced exercise tolerance and impaired haemodynamic function are seminal features of the heart failure (HF) syndrome.^1^ There is increasing awareness that comorbidities such as atrial fibrillation (AF) contribute strongly to symptoms, functional limitations, and clinical outcomes in individuals with HF^1^—including HF with reduced (HFrEF) and preserved ejection fraction (HFpEF). Atrial fibrillation is present in approximately 20–65% of patients with HF,^2^ making it the most common arrhythmia and particularly in older populations—a major HF comorbidity.^1,2^ This reflects shared risk factors and pathophysiology between the two conditions^2,3^ and the bi-directional impact of each condition on the other.^1,3^ As a result, there is a growing understanding that patients with both HF and AF may have a distinct HF subtype.^1^ However, the phenotypic features underlying the combined impact of HF and AF have not been definitively established.^1^

Therefore, we performed a systematic search and meta-analysis to quantify the impact of AF on crucial features of the HF syndrome, specifically exercise tolerance (measured objectively as peak oxygen uptake, VO_2_peak) and exercise haemodynamics.

## Methods

### Data sources and search strategy

PubMed, Scopus, and Web of Science databases were systematically searched to find articles published from inception to June 2024. Our search strategy focused on three key terms: (i) heart failure, (ii) atrial fibrillation, and (iii) cardiopulmonary exercise test (see Supplementary material online, *Methods* for full search terms). Additionally, we manually searched the reference lists of included studies using Google Scholar.

Articles identified from these databases were imported into the Covidence review management software (Melbourne, Australia). Initial screening involved screening titles and abstracts, followed by a full-text review against predefined inclusion criteria by two researchers (M.J.H., V.S.) with conflicts resolved by another independent researcher (S.J.F.). Following study inclusion, the relevant data of all included studies were extracted independently by the two researchers (M.J.H., V.S.) for synthesis. No ethical approval was required for this study since all data were sourced from previously published studies and did not involve any personally identifiable information.

### Study selection criteria

Only studies were included that: (i) examined participants with HF; (ii) compared subjects with AF with non-AF (i.e. sinus rhythm, SR); (iii) measured VO_2_peak from expired gas analysis. The exclusion criteria were: (i) no original or duplicate data; (ii) no HF; (iii) no SR comparator group; (iv) non-human cohorts (i.e animal models); (v) non-English studies; and (vi) no peak exercise data.

### Study quality assessment

Study quality and risk of bias were evaluated using the AXIS appraisal tool—a validated 20-point tool designed to assess the quality of cross-sectional studies (a maximum score of 20 indicates the highest quality).^4^

### Data synthesis and analysis

A meta-analysis was conducted using the R metacont package (R Core Team, 2016, R Foundation for Statistical Computing, Vienna, Austria) to compare differences in primary and secondary outcomes between patients with HF and AF (HF-AF) and those with non-Afib/SR (HF-SR). Fixed effects models were used to analyse the data. The weighted average effect size, represented as the weighted mean difference (WMD) with 95% confidence intervals (CI), was calculated for each outcome. Study weights were determined by the inverse of the variance within each study, with larger studies receiving higher weights due to their larger sample sizes. Heterogeneity was assessed using *I*^2^ and *τ*^2^ statistics. Forest plots were generated to visualize individual effect sizes, standard deviations, and the corresponding *P*-values (alpha = 0.05).

## Results

### Included studies

**Figure 1:**
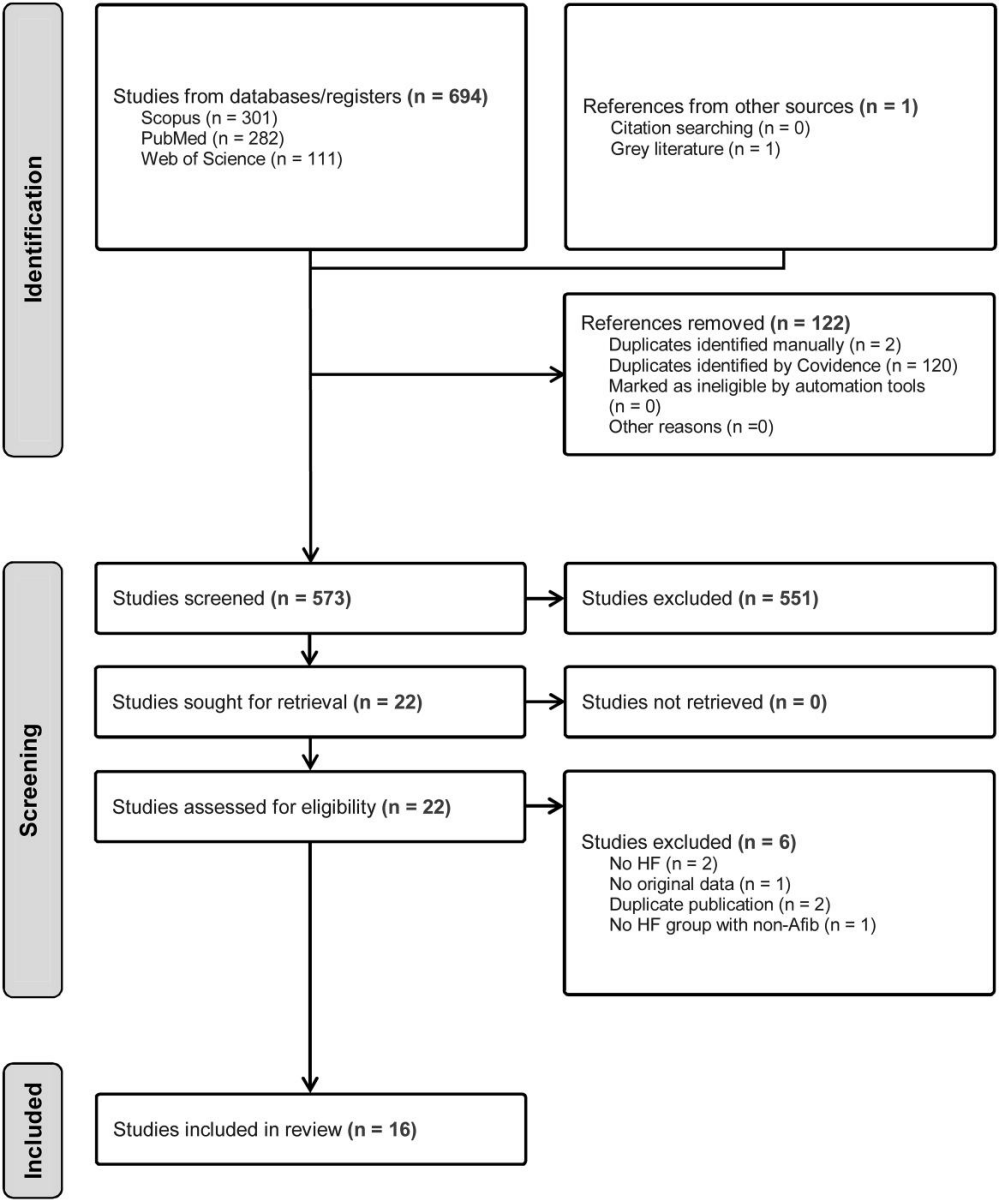
PRISMA diagram outlining the outcomes for the identification and screening of studies to be included in the meta-analysis.

After removal of duplicate studies, 573 studies were reviewed against the inclusion criteria (*Figure 1*), with 16 studies included (*Table 1*) ^5–20^ comparing VO_2_peak with or without exercise haemodynamics in HF-AF (*n* = 1,271, 68% male, age: 66.5 years, resting LVEF: 41.2%) or HF-SR (*n* = 4910; 62% male, age: 62.4 years, resting LVEF: 40.8%). Cohorts included HFrEF only, HFpEF only, or a combination of HF sub-types (*Table 1*). Peak oxygen uptake was assessed from a treadmill test in five studies, upright cycling in five studies, supine cycling in two studies, and a combination of either treadmill or supine cycling in four studies.

**Table 1:** Clinical, demographic and methodologic data for included studies

Author, year	HF-phenotype	Group	No./sex	Age	LVEF	Rate control medications			CPET mode
						*Beta-blocker*	*Digoxin*	*Amiodarone*	
Pardaens, 1997^5^	HFrEF	HF-AF	18 males	53 years	21%	11%	89%	*NR*	CYC (UR)
		HF-SR	93 males	50 years	23%	15%	67%	*NR*	
Pozzoli, 1998^6^	HFrEF	HF-AF	14 males, 4 females	54 years	24%	*NR*	*NR*	*NR*	TM
		HF-SR	255 males, 35 females	54 years	23%	*NR*	*NR*	*NR*	
Parthenakis, 2007^7^	HFrEF	HF-AF	29 males, 11 females	63 years	27%	68%	55%	15%	TM
		HF-SR	79 males, 28 females	57 years	30%	71%	21%	15%	
Agostoni, 2008^8^	Mixed (HFrEF + HFpEF)	HF-AF	148 males, 32 females	63 years	31%	64%	20%	18%	CYC (UR) or TM
		HF-SR	565 males, 197 females	58 years	32%	70%	29%	23%	
Waldenhjort, 2009^9^	HFrEF	HF-AF	26 males, 15 females	74 years	39%	68%	68%	NR	CYC (UR)
		HF-SR	18 males, 8 females	73 years	31%	46%	27%	NR	
Zakeri, 2014^10^	HFpEF	HF-AF	47 males, 32 females	73 years	59%	80%	23%	1%	CYC (UR) or TM
		HF-SR	57 males, 67 females	66 years	63%	73%	2%	6%	
Palmero, 2016^11^	HFrEF	HF-AF	11 males, 4 females	64 years	36%	100%	13%	20%	CYC (UR)
		HF-SR	11 males, 7 females	63 years	33%	94%	0%	44%	
Abreu, 2017^12^	HFrEF	HF-AF	28 males, 7 females	71 years	27%	NR	NR	NR	TM
		HF-SR	41 males, 25 females	67 years	26%	NR	NR	NR	
Elshazly, 2017^13^	HFpEF	HF-AF	154 males, 85 females	59 years	58%	69%	21%	NR	CYC (UR) or TM
		HF-SR	875 males, 630 females	50 years	60%	61%	5%	NR	
Kaye, 2017^14^	Mixed (HFmrEF + HFpEF^a^)	HF-AF	20 males/females	69 years	46%	65%	35%	NR	CYC (supine)
		HF-SR	35 male/females	70 years	48%	63%	3%	NR	
Lam, 2017^15^	HFpEF	HF-AF	12 males, 20 females	74 years	56%	81%	NR	NR	TM
		HF-SR	16 males, 46 females	73 years	58%	79%	NR	NR	
Luo, 2017^16^	HFrEF	HF-AF	382 males, 61 females	63 years	24%	91%	54%	85%	CYC (UR) or TM
		HF-SR	1602 males, 537 females	57 years	25%	95%	41%	81%	
Gonçalves, 2020^17^	HFrEF	HF-AF	45 males, 6 females	58 years	26%	78%	NR	NR	TM
		HF-SR	162 males, 61 females	53 years	29%	80%	NR	NR	
Reddy, 2020^18^	HFpEF	HF- AF	19 males, 29 females	75 years	61%	75%	23%	NR	CYC (supine)
		HF-SR	69 males, 112 females	66 years	64%	51%	1%	NR	
Chuda, 2021^19^	Mixed (HFrEF + HFpEF)	HF-AF	23 males/females	67 years	41%	87%	NR	NR	CYC (UR)
		HF-SR	23 males/females	67 years	46%	87%	NR	NR	
Palau, 2023^20^	HFpEF	HF-AF	30 males, 38 females	73 years	67%	88%	4%	NR	CYC (UR)
		HF-SR	28 males, 37 females	74 years	66%	89%	5%	NR	

Data are presented as mean unless otherwise specified.

AF, atrial fibrillation; CPET, cardiopulmonary exercise testing; CYC, cycling; HF, heart failure; LVEF, left ventricular ejection fraction; SR, sinus rhythm; TM, treadmill; UR, upright.

^a^HF-phenotype redefined according to the current guidelines (HFrEF ≤40% EF, HFmrEF 40–49% EF, HFpEF ≥50% EF)^21^.

### Peak oxygen uptake

**Figure 2:**
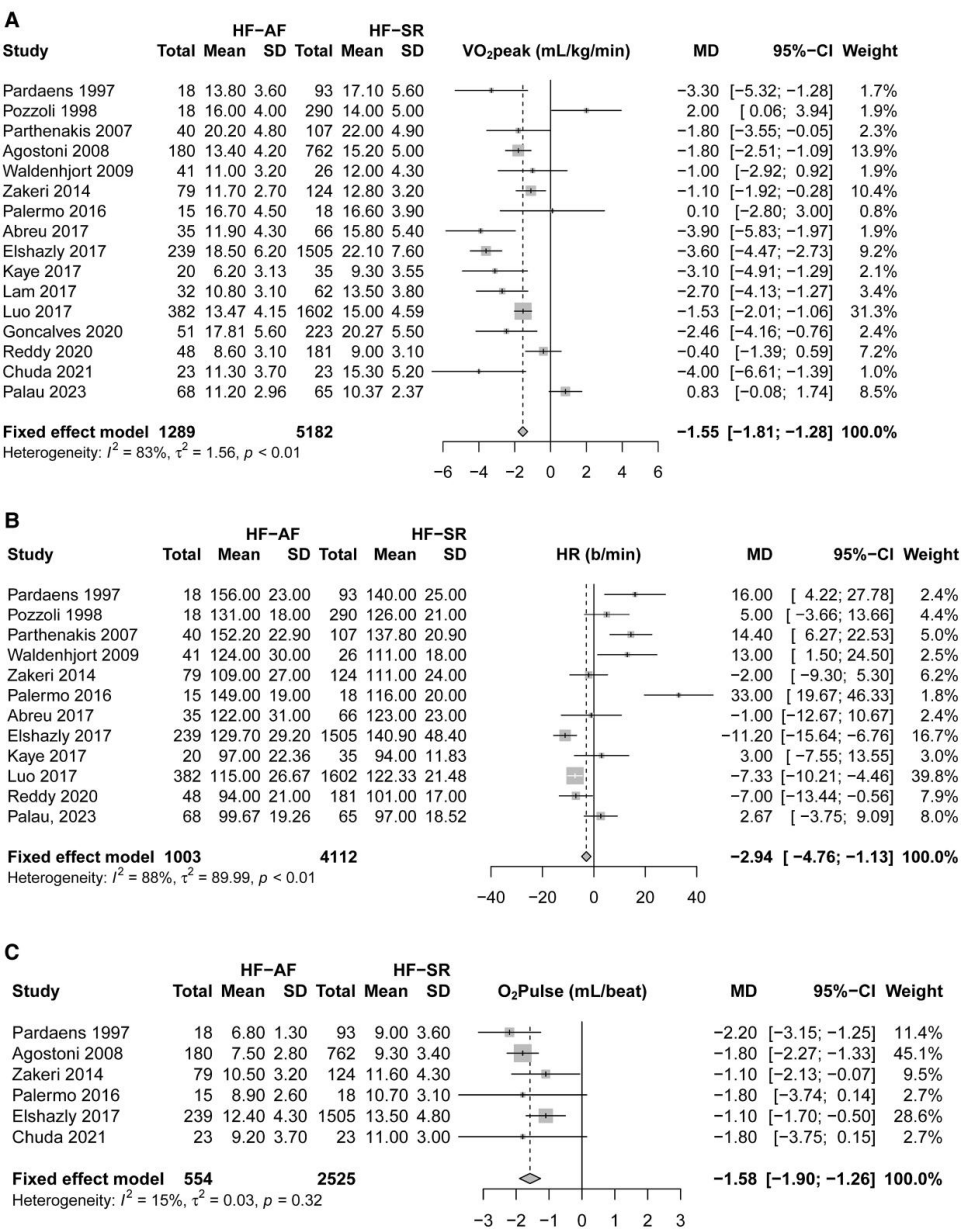
Impact of AF status on VO_2_peak and exercise haemodynamics in individuals with heart failure. Forest plots showing peak oxygen uptake indexed to bodyweight (*A*), peak exercise heart rate (*B*) and peak exercise oxygen pulse for individuals with heart failure with atrial fibrillation or in sinus rhythm. HR, heart rate; VO_2_peak, peak oxygen uptake; O_2_pulse, oxygen pulse; HF-AF, heart failure with atrial fibrillation; HF-SR, heart failure with sinus rhythm.

Body weight-indexed VO_2_peak was significantly lower in patients with HF-AF (14.01 mL/kg/min) compared with HF-SR (15.72 mL/kg/min; WMD: −1.55 mL/kg/min, 95% CI: −1.81 to −1.28 mL/kg/min, *I*^2^ = 83%, *n* = 6471; *Figure 2A*). This difference was also evident when measured in absolute values (WMD: −128.74 mL/min, 95% CI: −175.26 to −82.21 mL/min, *I*^2^ = 55%, *n* = 1328; Supplementary material online, *Figure S1A*). Meta-regression analysis showed no relationship between the mean resting left ventricular ejection fraction for each study and the MD for VO_2_peak in HF-AF (*ß* = −0.05, 95% CI: −0.19 to 0.09, *P* = 0.479) vs. HF-SR (*ß* = −0.05, 95% CI: −0.19 to 0.09, *P* = 0.462). Peak power output (WMD: −12.63 W, 95% CI: −16.56 to −8.69, *I*^2^ = 0%, *n* = 1311; Supplementary material online, *Figure S2B*) was also significantly lower in HF with AF than in HF-SR.

### Exercise haemodynamics

The lower VO_2_peak in HF-AF coincided with a marginally lower peak exercise heart rate (HR; WMD: −2.94 b/min, 95% CI: −4.76 to −1.13 b/min, *I*^2^ = 88%, *n* = 5115; *Figure 2B*) and a lower peak exercise O_2_pulse (WMD: −1.58 mL/beat, 95% CI: −1.90 to −1.26 mL/beat, *I*^2^ = 15%, *n* = 3049; *Figure 2C*). Peak exercise systolic blood pressure (SBP) was also lower in HF-AF relative to HF-SR (WMD: −11.11 mmHg, 95% CI: −14.01 to −8.21 mmHg, *I*^2^ = 69%, *n* = 2409; Supplementary material online, *Figure S2*). Only two studies^14,18^ measured stroke index, cardiac index, and arterio-venous oxygen difference (a-vO_2_diff), and so meta-analysis was not performed for these outcomes. In both studies, stroke index (MD: −12.00 mL/m^2^, 95% CI: −17.54 to −6.46 mL/m^214^; MD: −9.00 mL/m^2^; 95% CI: −13.33 to −4.67 mL/m^218^) and cardiac index (MD: −0.90 L/min/m^2^, 95% CI: −1.45 to −0.35 L/min/m^214^; MD: −1.40 L/min/m^2^, 95% CI: −1.76 to −1.04 L/min/m^218^) were significantly lower in HF-AF vs. HF-SR. The a−vO_2_diff was significantly higher in HF-AF in Reddy *et al*.^18^ (MD: 1.80 mL/dL, 95% CI: 1.16 to 2.44 mL/dL), but lower in HF-AF in the study of Kaye *et al*.^14^ (albeit not significant; MD: −1.50 mL/dL, 95% CI: −3.19 to 0.19 mL/dL).

### Study quality and risk of bias

The mean AXIS score of the included studies was 15.3 points, suggesting a moderate level of bias (see Supplementary material online, *Table S1*).

## Discussion

To the best of our knowledge, this is the first systematic review and meta-analysis to quantify the impact of AF on VO_2_peak and exercise haemodynamics in patients with HF. In doing so, we made two key findings: (i) AF exacerbates exercise limitations in HF, such that VO_2_peak was 1.6 mL/kg/min (∼12%) lower in HF-AF than HF-SR, and this difference was consistent across the LVEF spectrum; (ii) the lower VO_2_peak in individuals with HF-AF is not the result of peak HR differences (which was marginally lower in HF-AF), but is likely due to ventricular and/or peripheral impairments.

The findings of our meta-analysis confirm that AF imposes additional and meaningful limitations to exercise tolerance beyond HF alone. Results from the HF-ACTION trial suggest the magnitude of VO_2_peak impairment in the HF-AF cohort (1.6 mL/kg/min lower) is clinically significant, as every 1.0 mL/kg/min decrement in VO_2_peak has been associated with an ∼15% increase in all-cause mortality.^22^ Moreover, the mean VO_2_peak values in both AF and SR groups were below the threshold for independent living^23^ (15–18 mL/kg/min), where even small decrements in VO_2_peak can result in basic daily activities (walking, carrying groceries) exceeding their maximum capacity. Taken together, the lower VO_2_peak in HF-AF provides an explanation for greater functional limitations, reduced quality of life and worse clinical outcomes in individuals with HF and AF.^1^ This highlights the importance of AF-targeted therapies in patients with HF. Specifically, rhythm control interventions such as AF ablation,^24,25^ and lifestyle interventions targeting risk factors for AF progression^26,27^ which are associated with improved exercise tolerance, haemodynamic function, and arrhythmia burden.

By investigating the underlying mechanisms contributing to functional limitations in HF-AF, we demonstrated that the reduced VO_2_peak is not mediated by differences in peak HR, which was marginally lower (∼3.5 b.p.m. lower) in those with AF. Based on the Fick principle, this suggests that the lower VO_2_peak in HF-AF was primarily due to lower stroke volume (SV) and/or a-vO_2_diff. Unfortunately, there were insufficient studies to quantify either outcome in our meta-analysis. However, the two studies evaluating exercise SV responses reported stroke index and cardiac index were significantly lower in HF-AF vs. HF-SR.^14,18^ Moreover, the lower O_2_pulse (1.58 mL/beat or ∼15% lower) and peak SBP (∼10 mmHg lower) in the HF-AF cohorts suggest impairment in SV and/or a-vO_2_diff. The atrial mechanisms contributing to the lower SV could be impaired ventricular filling from the loss of atrial kick or beat-to-beat variations in filling time.^1^ Microvascular and skeletal muscle dysfunction may also decrease VO_2_peak in AF via their impact on the a-vO_2_diff and muscle oxygen diffusive conductance. Whilst pathophysiologic drivers of AF (e.g. chronic low-grade inflammation, hypertension^1^) could impair muscle-microvascular function, the two studies evaluating the a-vO_2_diff in HF-AF vs. HF-SR report conflicting results—reporting a-vO_2_diff is either higher in HF-AF^18^ (suggesting AF is primarily a cardiac limitation) or lower^14^ (suggesting a mixed cardiac-peripheral limitation). Therefore, further work is needed to understand the mechanisms contributing to decreased VO_2_peak in patients with HF and AF.

Notable limitations include significant heterogeneity among the included studies, which may reflect differences in assessment modalities, AF and HF aetiology, rate control medications, and participant characteristics (e.g. age, sex). This variability may have influenced the pooled results and should be considered when interpreting the findings. We also cannot rule out that a subset of patients in the HF-SR cohorts may have paroxysmal AF and/or sub-clinical atrial myopathy that has not progressed to persistent or permanent AF. However, this would attenuate differences between the HF-AF and HF-SR cohorts. As noted above, there were also insufficient studies to quantify the impact of AF on key exercise haemodynamic measures. Additionally, as assessed by the AXIS tool, moderate bias exists in the included studies, which could impact the reliability and generalizability of the findings. Future research with standardized protocols and rigorous designs is needed to address these limitations.

## Conclusions

In patients with HF across the spectrum of LVEF, AF is associated with clinically meaningful reductions in VO_2_peak, potentially due to reduced SV and/or arterio-venous oxygen difference. This association highlights the importance of identifying and managing major comorbidities such as AF in individuals with HF.

## Supplementary Material

oeaf003_Supplementary_Data

## Data Availability

The data used in this analysis are obtained from publicly accessible sources. All original data and studies are available in the references cited within the manuscript and the supplementary materials.
