# Supplementary material for: The impact of atrial fibrillation on oxygen uptake and haemodynamics in patients with heart failure: a systematic review and meta-analysis
Source: Eur Heart J Open. 2025 Feb 4;5(1):oeaf003. doi: 10.1093/ehjopen/oeaf003 (PMC11823826; doi:10.1093/ehjopen/oeaf003)
Supplement: oeaf003_Supplementary_Data [file oeaf003_supplementary_data.pdf]

Supplemental Materials

Title: The impact of atrial fibrillation on oxygen uptake and haemodynamics in patients with heart failure: A systematic review and meta-analysis

Authors: Veronika Schmid[1,2]*, Stephen J Foulkes[2,3*], Jenelle K Dziano[4], Jing Wang[5], Jan Verwerft,[6,7] Adrian D Elliott[4], Mark J Haykowsky[2]

[1] Technical University of Munich, School of Medicine and Health, Department for Preventive Sports Medicine and Sports Cardiology, TUM University Hospital, Germany.

[2] Integrated Cardiovascular Exercise Physiology and Rehabilitation Lab, Faculty of Nursing, College of Health Sciences, University of Alberta, Edmonton, Alberta, Canada.

[3] Heart, Exercise and Research Trials Lab, St Vincent's Institute of Medical Research, Fitzroy, Victoria, Australia.

[4] Centre for Heart Rhythm Disorders, University of Adelaide & Royal Adelaide Hospital, Adelaide, South Australia, Australia.

[5] Division of Public Health, School of Medicine, University of Utah, Salt Lake City, Utah, USA.

[6] Department of Cardiology, Jessa Hospital, Hasselt, Belgium.

[7] Faculty of Medicine and Life Sciences, UHasselt, Diepenbeek, Belgium.

*Contributed equally as first authors.

Supplementary Methods

Search Terms:

Pubmed:

- Term: ("exercise test"[MeSH Terms] OR ("exercise"[All Fields] AND "test"[All Fields]) OR "exercise test"[All Fields] OR ("cardiopulmonary"[All Fields] AND "exercise"[All Fields] AND "test"[All Fields]) OR "cardiopulmonary exercise test"[All Fields]) AND ("heart failure"[MeSH Terms] OR ("heart"[All Fields] AND "failure"[All Fields]) OR "heart failure"[All Fields]) AND ("atrial fibrillation"[MeSH Terms] OR ("atrial"[All Fields] AND "fibrillation"[All Fields]) OR "atrial fibrillation"[All Fields])

<u>Translations:</u>

cardiopulmonary exercise test: "exercise test"[MeSH Terms] OR ("exercise"[All Fields] AND "test"[All Fields]) OR "exercise test"[All Fields] OR ("cardiopulmonary"[All Fields] AND "exercise"[All Fields] AND "test"[All Fields]) OR "cardiopulmonary exercise test"[All Fields]

heart failure: "heart failure"[MeSH Terms] OR ("heart"[All Fields] AND "failure"[All Fields]) OR "heart failure"[All Fields]

Atrial fibrillation: "atrial fibrillation"[MeSH Terms] OR ("atrial"[All Fields] AND "fibrillation"[All Fields]) OR "atrial fibrillation"[All Fields]

- Results: 282 (Date: 05/June/2024)
- Years: 1977-2024

Scopus:

- Term: Edit

(ALL ("cardiopulmonary exercise test") AND TITLE-ABS-KEY ("heart failure") AND TITLE-ABS-KEY ("atrial fibrillation"))

- Results: 301 (Date: 05/June/2024)
- Years: 1993-2024

Web of Science:

- Term:

((ALL=(cardiopulmonary exercise test)) AND ALL=(heart failure)) AND ALL=(Atrial fibrillation)

- Results: 111 (Date: 05/June/2024)
- Years: 1995-2024

Supplementary Figures

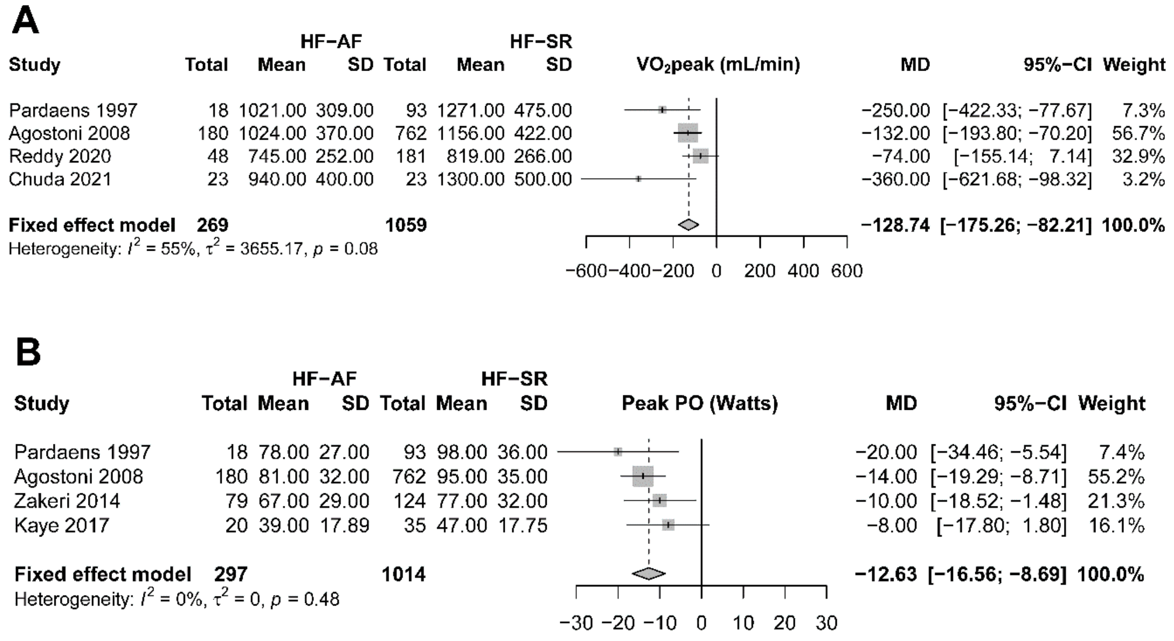

Supplementary Figure 1. Impact of AF status on absolute VO_2peak and peak power output in individuals with heart failure. Forest plots showing absolute VO_2peak (mL/min; Panel A) and peak power output (PO, Watts; Panel B) for individuals with heart failure with atrial fibrillation (HF-AF) or in sinus rhythm (HF-SR).

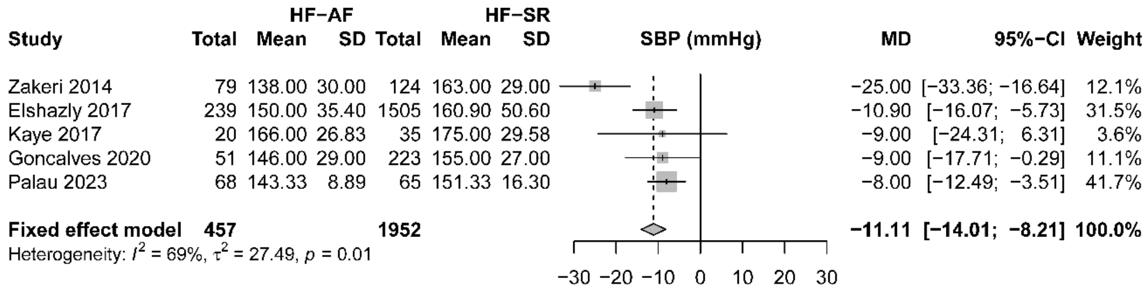

Supplementary Figure 2. Impact of AF status peak exercise systolic blood pressure in individuals with heart failure. Forest plots showing peak exercise systolic blood pressure (SBP, mmHg) for individuals with HF-AF or HF-SR.

Supplementary Table 1. Results of study quality assessment using the Axis Tool.

	**Pardaens, 1997**	**Pozzoli, 1998**	**Parthenakis, 2007**	**Agostoni, 2008**	**Waldenhjort, 2009**	**Zakeri, 2014**	**Palmero, 2016**	**Abreu, 2017**	**Elshazly, 2017**	**Kaye, 2017**	**Lam, 2017**	**Luo, 2017**	**Gonçalves, 2020**	**Reddy, 2020**	**Chuda, 2021**	**Palau, 2023**	
1. Were the aims/objectives of the study clear?	**1**	**1**	**1**	**1**	**1**	**1**	**1**	**1**	**1**	**1**	**1**	**1**	**1**	**1**	**1**	**1**	
2. Was the study design appropriate for the stated aim(s)?	**1**	**1**	**1**	**1**	**1**	**1**	**1**	**1**	**1**	**1**	**1**	**1**	**1**	**1**	**1**	**1**	
3. Was the sample size justified?	**0**	**0**	**0**	**0**	**0**	**0**	**0**	**0**	**0**	**0**	**0**	**0**	**0**	**0**	**0**	**0**	
4. Was the target/reference population clearly defined? (Is it clear who the research was about?	**1**	**1**	**1**	**1**	**1**	**1**	**1**	**1**	**1**	**1**	**1**	**1**	**1**	**1**	**1**	**1**	
5. Was the sample frame taken from an appropriate population base so that it closely represented the target/reference population under investigation?	**1**	**1**	**1**	**1**	**1**	**1**	**0**	**1**	**1**	**1**	**1**	**1**	**1**	**1**	**1**	**1**	
6. Was the selection process likely to select subjects/ participants that were representative of the target/ reference population under investigation?	**1**	**0**	**1**	**1**	**1**	**1**	**1**	**1**	**0**	**1**	**1**	**1**	**1**	**1**	**1**	**1**	

7. Were measures undertaken to address and categorize non-responders?	**0**	**0**	**0**	**0**	**0**	**0**	**0**	**0**	**0**	**0**	**0**	**0**	**0**	**0**	**0**	**0**	
8. Were the risk factor and outcome variables measured appropriate to the aims of the study?	**1**	**1**	**1**	**1**	**1**	**1**	**1**	**1**	**1**	**1**	**1**	**1**	**1**	**1**	**1**	**1**	
9. Were the risk factor and outcome variables measured correctly using instruments/ measurements that had been trialled, piloted or published previously?	**1**	**1**	**1**	**1**	**1**	**1**	**1**	**1**	**1**	**1**	**1**	**1**	**1**	**1**	**1**	**1**	
10. Is it clear what was to determined statistical significance and/or precision estimates? (e.g. P values CIs)	**1**	**1**	**1**	**1**	**1**	**1**	**1**	**1**	**1**	**1**	**1**	**1**	**1**	**1**	**1**	**1**	
11. Were the methods (including statistical methods) sufficiently described to enable them to be repeated?	**1**	**1**	**1**	**1**	**1**	**1**	**1**	**1**	**1**	**1**	**1**	**1**	**1**	**1**	**1**	**1**	
12. Were the basic data adequately described?	**1**	**1**	**1**	**1**	**1**	**1**	**1**	**1**	**1**	**1**	**1**	**1**	**1**	**1**	**1**	**1**	

13. Does the response rate raise concerns about non-response bias?	**0**	**0**	**0**	**0**	**0**	**0**	**0**	**0**	**0**	**0**	**0**	**0**	**0**	**0**	**0**	**0**	
14. If appropriate, was information about non-responders described?	**0**	**0**	**0**	**0**	**0**	**0**	**0**	**0**	**0**	**0**	**0**	**0**	**0**	**0**	**0**	**0**	
15. Were the results internally consistent?	**0**	**1**	**1**	**1**	**1**	**1**	**1**	**1**	**1**	**1**	**1**	**1**	**1**	**1**	**1**	**1**	
16. Were the results for the analyses described in the methods, presented?	**1**	**1**	**1**	**1**	**1**	**1**	**1**	**1**	**1**	**1**	**1**	**1**	**1**	**1**	**1**	**1**	
17. Were the author´s discussions and conclusion justified by the results?	**1**	**1**	**1**	**1**	**1**	**1**	**1**	**1**	**1**	**1**	**1**	**1**	**1**	**1**	**1**	**1**	
18. Were the limitations of the study discussed?	**0**	**1**	**1**	**0**	**1**	**1**	**0**	**1**	**1**	**1**	**1**	**1**	**1**	**1**	**1**	**1**	
19. Were there any funding sources or conflicts of interest that may affect the authors interpretation of the results?	**0**	**1**	**1**	**1**	**1**	**1**	**1**	**1**	**1**	**1**	**1**	**1**	**1**	**1**	**1**	**1**	
20. Was ethical approval or consent of participants attained?	**0**	**0**	**1**	**0**	**1**	**1**	**1**	**1**	**1**	**1**	**1**	**1**	**1**	**1**	**1**	**1**	
**Score**	**12**	**14**	**16**	**14**	**16**	**16**	**14**	**16**	**15**	**16**	**16**	**16**	**16**	**16**	**16**	**16**	**15.3**
